# Assessment of Mechanical Properties for Three-Dimensional Needled Composites: A Geometric Partitioning Strategy Dealing with Mesoscopic Needling Damage

**DOI:** 10.3390/ma15165659

**Published:** 2022-08-17

**Authors:** Rongqiao Wang, Yu Liu, Xi Liu, Dianyin Hu, Jiangbo Han, Penghui Ma, Xiaojun Guo

**Affiliations:** 1 School of Energy and Power Engineering, Beihang University, Beijing 100191, China; 2 Research Institute of Aero-Engine, Beihang University, Beijing 100191, China; 3 Beijing Key Laboratory of Aero-Engine Structure and Strength, Beijing 100191, China; 4 United Research Center of Mid-Small Aero-Engine, Beijing 100191, China; 5 Hunan Aviation Powerplant Research Institute, Aero Engine Corporation of China, Zhuzhou 412002, China

**Keywords:** the general method of cells model, continuum damage mechanics model, needled composite, geometric partitioning strategy

## Abstract

A geometric partitioning strategy was proposed to evaluate the mechanical properties of three-dimensional needled composites. The microstructure of the composite was divided to accurately characterize the mesoscopic damage in the needling regions and the macroscopic damage in the un-needling regions, to balance the computational accuracy and efficiency. The general method of cells (GMC) models along with the damage criteria were established for different material phases in the needling regions, while the continuum damage mechanics (CDM) model was adopted to portray the damage evolution in the un-needling regions. Through conducting the multi-scale simulation, the mechanical properties of the needled composites were predicted, based upon which the effect of repeated needling on the mesoscale damage process was further investigated. Results showed that the predictions are in good agreement with the experiments, with a relative error of 2.6% for strength and 4.4% for failure strain. The proposed approach can provide guidance for the process optimization and design of needled composites.

## 1. Introduction

Carbon fiber-reinforced ceramic matrix composites (carbon/silicon carbide, C/C-SiC) have gained wide attentions due to their excellent mechanical properties, high temperature resistance, and superior friction properties [1,2,3]. In order to match the usage requirements of various structures, different composite forms have been developed such as laminates [4] and multi-dimensional weaving [5]. Among them, the three-dimensional (3D) needled composite, which is processed by introducing in-plane fibers into the thickness direction through the needling process, is superior in its interlayer properties and simple process. Currently, the needled composites have been widely utilized in braking systems, rocket engine nozzle throats and exit cones [6].

Needled composites can be geometrically divided into the needling regions and the un-needling regions. Since the needled composites are composed of multiple material phases, it is difficult to model and simulate the mechanical properties. Based on the well-developed laminate theory, several analytical approaches have been deployed to simulate the mechanical properties, including the methods of characteristic variables [7], inclusions equivalence [8], and irregular beams model [9]. However, modified theoretical methods are usually associated with long periods and high costs in conjunction with extensive basic experiments to determine the model parameters. Furthermore, these approaches are generally based on empirical mixture rules, failing to reflect the failure mechanisms of needled composites [10,11]. In contrast, finite element (FE) methods are more promising for complex geometrical analysis [12]. Alternately, the nonlinear damage constitutive theories have been proposed on the macroscopic scale. For instance, Xie et al. [13] investigated the mechanical behavior of needled composites based on the elastoplastic theory of metals. Despite the fact that the model exhibits some abilities in the damage characterization, it has limitations especially considering that the progressive damage of composites does not involve a distinct yielding stage [14]. Inspired by this, the continuum damage mechanics (CDM) model was developed as a flexible tool for strength analysis of unidirectional plies [15], laminates [16], and braided composites [17], by introducing the damage tensor to depict the anisotropic damage. From the framework of thermodynamics, the damage equation constructed based on the principle of minimum dissipation has the capability of reflecting the macroscopic damage mechanism of the composites. Therefore, the CDM model is introduced in this work to accurately portray the nonlinear damage behavior of the un-needling regions.

For the needling regions, the needling process inevitably results in mesoscopic fiber damage, thus degrading the material strength [18]. Moreover, the needling fibers in the thickness direction transform the material from a planar periodic to a 3D non-periodic structure. In this condition, the CDM model is no longer applicable, since it cannot express the local micromechanical characteristics [19]. Meanwhile, experiments [20,21,22] showed that the stress concentration regions around the needling holes would generate the local mesoscopic damage, which would eventually evolve into the structural failure. Thus, the mechanical analysis of needled composites should focus on the damage evolution of the mesoscopic structure in the needling regions. In this field, Irene et al. [23,24] derived the stress and displacement redistributions based on the shear-lag theory for a continuous *N*-fiber model in unidirectional fibers with an internal notch. Further, Qi et al. [25] establish the relationship between the stress distribution and needling parameters through a surrogate model in an attempt to guide the material process. However, shear-lag model as a static analytical method is unable to predict the microstructural evolution of the material under load, which is the mainly failure characteristic of needled composites [26]. At the same time, the complex composition of needled composites leads to multiparameter, high-dimensional nonlinear analysis, which is not satisfactorily calculated by surrogate models considering only a single material phase layer [27]. To address these issues, it is recommended to develop the separate numerical models for different material phases from the real microstructure, aiming to accurately characterize the mesoscopic damage behavior in the needling region.

On the other hand, since the degradation of material properties at the mesoscopic scale would affect the macroscopic field variables, it is requisite to establish a dynamic transfer relationship between data at different scales. In this regard, Aboudi [28] proposed the general method of cells (GMC) to achieve bidirectional transfer of parameters across scales by establishing a relational equation between macroscopic and mesoscopic field quantities. Guided by this thought, NASA implemented the software by combining the GMC program and FE method [29], which has been applied to the analysis of open-hole laminates [30,31]. Currently, the GMC model is mainly applied to analyzing the fiber bundles in unidirectional [32] and woven composites [33]. The needled region, however, has the needling holes and needling fibers in addition to the long fibers and matrix, which increases the difficulty of GMC modelling and subcells partitioning. Therefore, to the authors’ knowledge, the GMC model has not been extended to needled composites. However, in order to accurately characterize the damage processes and failure mechanisms of needled composites, GMC models for each material phase are required to enable multi-scale analysis. Nevertheless, the computational cost of multi-scale is clearly higher than that of CDM due to the mesoscale calculations at each integration point. Thus, in order to accurately characterize the mesoscopic damage evolution of the needling regions while maintaining the computational efficiency, it is urgent to apply a geometric partitioning strategy in which the needling regions are described by the multi-scale method and the un-needling regions are covered through the CDM model.

In this context, mesoscopic scale GMC models are constructed for 3D needled composites with different material phases. A geometric partitioning strategy is proposed to evaluate the mechanical properties of the needled composites: a multi-scale method is established to simulate the mesoscale damage evolution in the needling regions, and the CDM model is introduced for characterizing the failure process in the un-needling regions. The work is constructed as follows: In Section 2, the materials and needling processes are described in detail. The geometric partitioning strategy for the needling/un-needling regions is developed in Section 3, together with the respective damage models. Section 4 presents the experimental results and discussions. Finally, conclusions are drawn in Section 5.

## 2. Materials

**Figure 1:**
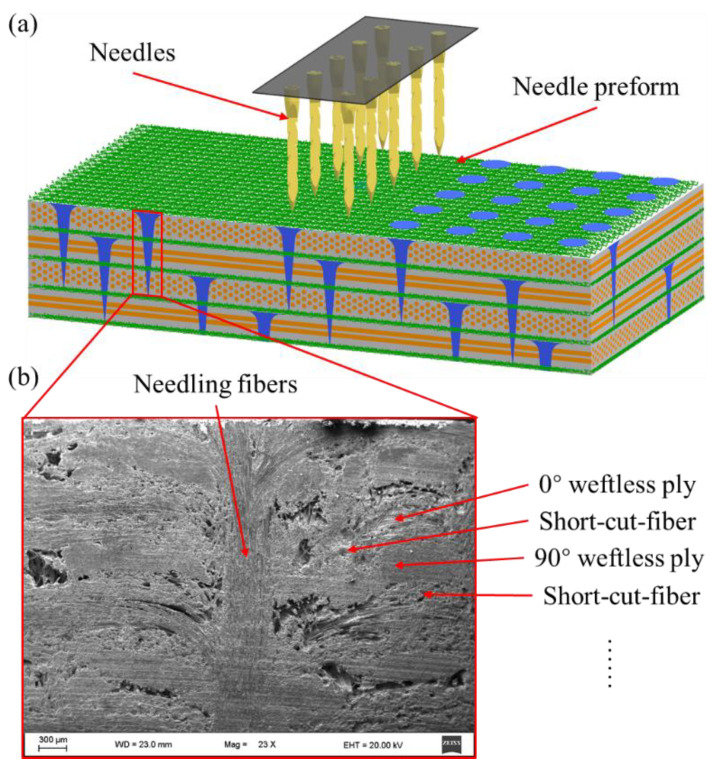
(**a**) Schematic illustration of the needling process (**b**) scanning electron microscope (SEM) image of the needling region.

The 3D needled composites in this paper were provided by the Institute of Metals Research, Chinese Academy of Sciences [34], in which the needling density and needling depth are 24.96 punches/cm^2^ and 3 mm, respectively. During the needling process, the unique structural features are created, as shown in Figure 1. The needling process contains two steps: 0° weftless ply, short-cut fiber, 90° weftless ply, and short-cut fiber are laid alternately back and forth to shape the needle-punching preform in the first step. The second step is to punch the needles vertically into the stacked plies after placing each layer of short-cut fibers. These above two steps are duplicated until a given thickness is achieved, resulting in a formed preform.

The material parameters of the fibers and the matrix are listed in Table 1, in which the porosity is obtained by threshold segmentation of the micro-computed tomography (μCT) images [35]. The μCT utilized in this study is conducted with the nanoVoxel equipment provided by Sanying Precision Instruments Co., Ltd., Tianjin, China.

## 3. Numerical Simulation Theory and Method

### 3.1. Mesoscopic Model

#### 3.1.1. Needling Regions Modelling

**Figure 2:**
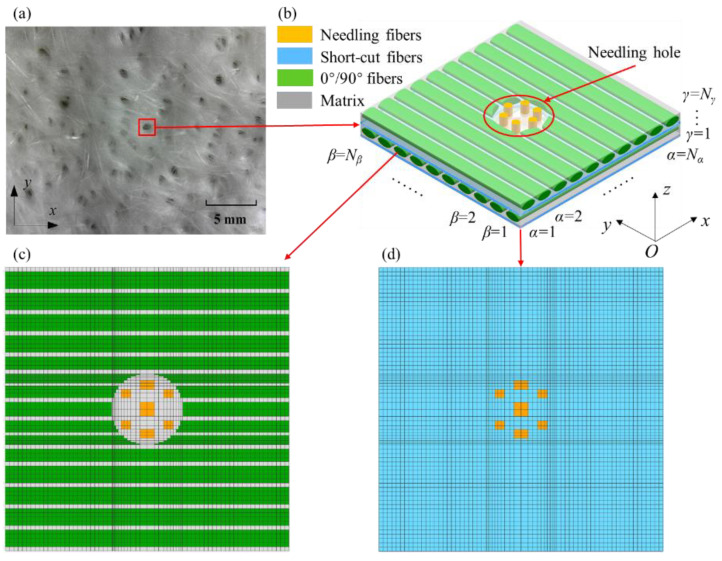
(**a**) Needling holes on the preform [37] (**b**) schematic diagram of a needling hole structure (**c**) GMC model of 0°/90° weftleshfps plies with a needling hole (**d**) GMC model of short-cut fibers with a needling hole.

**Figure 3:**
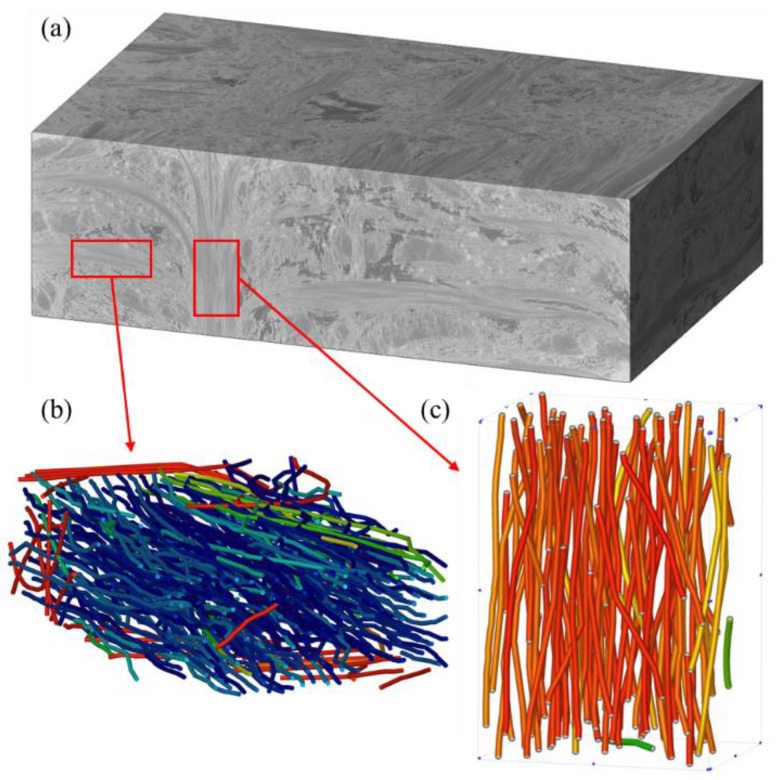
(**a**) μCT image (**b**) fiber distribution of unidirectional fibers and (**c**) needling fibers.

The needling process left needling holes on the preforms, as shown in Figure 2a. Due to the repeated needling and the rotation of the preform, the needling holes are distributed densely and disorderly. Figure 2b depicts the mesoscopic model of the needling region, including a layer of 0° weftless ply, a layer of 90° weftless ply, and two layers of short-cut fibers, ensuring periodicity in the thickness direction. The radius of the needling hole and the unidirectional fibers are determined by their corresponding volume fractions, which are measured by the μCT. The scanning sample size is 6.57 × 9.87 × 3.50 mm. The local distributions of needling and unidirectional fibers in the needling region are presented in Figure 3, with the average local volume fractions of 18.7% and 76.2% for multiple measurement points, respectively. As a result, the GMC models are employed to establish the mesoscopic model of the needling regions. Each model is divided into *N_α_* × *N_β_* × *N_γ_* subcells. In Figure 2, *O*–*xyz* denotes the global coordinate system, in which *α*, *β*, and *γ* signify the subcell numbers along the *x*, *y*, and *z* directions, respectively.

In the *x*−*y* plane, the model is divided into 72 × 72 = 5184 subcells by considering the computational efficiency and the fineness of GMC geometry division, as shown in Figure 2c,d. *N*_0*α*_ = *N*_90*α*_ = *N_Sα_* = 72, *N*_0*β*_ = *N*_90*β*_ = *N_Sβ_* = 72, where the subscripts 0, 90, and *S* indicate 0° weftless plies, 90° weftless plies, and short-cut fiber plies, respectively. Due to the uniform random distribution of fibers, the short-cut fiber layers are assumed to be isotropic [7,38,39]. After the SEM observation of the thickness in each layer, *N*_0*γ*_ = *N*_90*γ*_ = 4 and *N_Sγ_* = 1 are measured.

The needling hole in the GMC model shows a stepped shape, whereas the FE model has a smooth transition, which is generally preferred by scholars. However, the microscopic observations demonstrated that the needling holes appeared irregular and rough [9,40], which is primarily caused by the non-circular needle cross-section and the pores in the matrix. The geometric conformity and mesoscopic structure characterization ability of the GMC model are examined in Reference [41], which also indicated that the GMC model is consistent with the FE model in simulating the mesoscopic stress distribution.

#### 3.1.2. Mesoscopic Damage Characterization

The damage models are established for the fiber and the matrix, respectively. Carbon fibers are a type of transversely isotropic material, where the Hashin criterion [42] is commonly utilized. The Hashin criterion includes four failure modes: the longitudinal tension (longitudinal in the fiber direction and transverse perpendicular to the fiber direction), the longitudinal compression, the transverse tension, and the transverse compression. The corresponding static strength expressions are shown in Equations (1)–(4). *X* and *Y* represent the strength in the longitudinal and transverse directions, respectively, while the superscripts “*T*” and “*C*” indicate the tension and compression, respectively.

Damage variables for the longitudinal tension *D*_1*T*_ (*σ*_11_ ≥ 0) [33]:

(1) $$D_{1T}={\left(\frac{\sigma_{11}}{X^{T}}\right)}^{2}+\frac{\alpha_{1}}{{\left(S^{L}\right)}^{2}}\left(\sigma_{12}^{2}+\sigma_{13}^{2}\right),$$

where *σ_ij_* (*i*, *j* = 1,2,3) represents the Cauchy stress component, while *S^L^* denotes the longitudinal shear strength.

Damage variables for the longitudinal compression *D*_1*C*_ (*σ*_11_ < 0) [33] is:

(2) $$D_{1C}={\left(\frac{\sigma_{11}}{X^{C}}\right)}^{2}.$$

Damage variables for the transverse tension *D*_23*T*_ (*σ*_22_ + *σ*_33_ ≥ 0) [33] is:

(3) $$D_{23T}={\left(\frac{\sigma_{22}+\sigma_{33}}{Y^{T}}\right)}^{2}+\frac{\alpha_{2}}{{\left(S^{L}\right)}^{2}}\left(\sigma_{12}^{2}+\sigma_{13}^{2}\right)+\frac{\alpha_{3}}{{\left(S^{T}\right)}^{2}}\left(\sigma_{23}^{2}-\sigma_{22}\sigma_{33}\right),$$

where *S^T^* expresses the transverse shear strength

Damage variables for the transverse compression *D*_23*C*_ (*σ*_22_ + *σ*_33_ < 0) [33] is:

(4) $$D_{23C}=\left[{\left(\frac{Y^{C}}{2S^{T}}\right)}^{2}-1\right]\frac{\sigma_{22}+\sigma_{33}}{Y^{C}}+{\left(\frac{\sigma_{22}+\sigma_{33}}{2S^{T}}\right)}^{2}+\frac{\alpha_{2}}{{\left(S^{L}\right)}^{2}}\left(\sigma_{12}^{2}+\sigma_{13}^{2}\right)+\frac{\alpha_{3}}{{\left(S^{T}\right)}^{2}}\left(\sigma_{23}^{2}-\sigma_{22}\sigma_{33}\right).$$

For the ceramic matrix, the Stassi criterion is adopted in view of the brittle nature [43,44], which is expressed as [45]:

(5) $$D_{m}=-3p\left[\frac{1}{X_{m}^{T}}-\frac{1}{X_{m}^{C}}\right]+\sigma_{\mathrm{von}}^{2}\frac{1}{X_{m}^{T}X_{m}^{C}},$$

where *D_m_* is the matrix damage parameter, $D_{m}\in \left[0,1\right]$. $X_{m}^{T}$ and $X_{m}^{C}$ stand for the tensile strength and the compressive strength, respectively, while *p* and *σ*_von_ represent hydrostatic pressure and the mises stress, respectively. The Stassi criterion enables the evaluation of the strength properties of inorganic compounds due to the asymmetry of the tensile and compressive mechanical behavior. Therefore, when $X_{m}^{T}=X_{m}^{C}$, the Stassi criterion devolves to the form of the von mises criterion.

To define the degradation of fiber properties after damage initiation, a linear-exponential damage model considering the characteristic element length is applied to accurately characterize the material softening [46]. Meanwhile, the Duvaut–Lions regularization model [47] is introduced to improve the convergence of the numerical algorithm. As the matrix are not the main components of the composite load-bearing, the stiffness reduction method is employed to describe its property degradation.

### 3.2. Macroscopic Model

#### 3.2.1. FE Modelling

**Figure 4:**
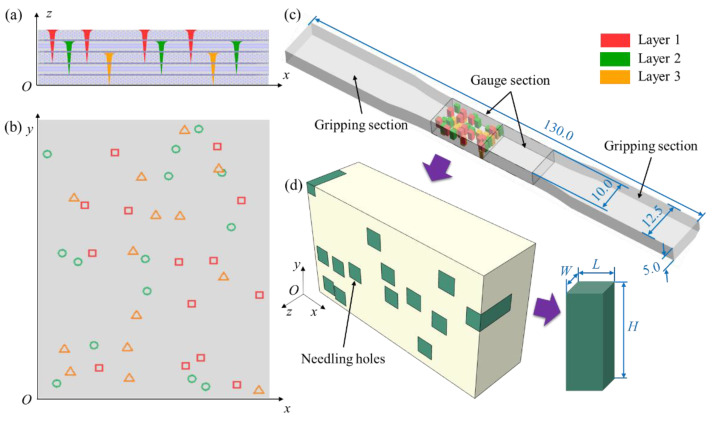
Schematic diagrams of (**a**) needling depth (*O*−*xz*) and (**b**) needling distribution (*O*−*xy*). (**c**) specimen size and model sampling location (unit: mm) (**d**) FE model.

Due to the dense and disorganized distribution of needling holes in the needled composites, a random sampling method is conducted to simulate the real distribution, with the side and top view schematics shown in Figure 4a, b, respectively. As depicted in Figure 4c, considering that the damage and failure of the specimen mainly occur in the gauge section, the gripping sections are ignored in the model. In order to further improve the calculation efficiency, half of the gauge section is taken to build the geometric model with the size of 17 × 10 × 5 mm. Referring to Reference [27], the final established FE model is shown in Figure 4d. The needling regions are macroscopically represented by rectangles (marked in green), whose planar dimensions are obtained by averaging multiple measurements from SEM observations. The length *L* and width *W* are both taken to be 1.34 mm, while the height *H* is the same as the needling depth.

#### 3.2.2. CDM Model of Un-Needling Regions

A CDM model proposed by Gao et al. [48] is introduced to simulate the stress-strain behavior of the un-needling regions, in which the damage behavior is driven by thermodynamic conjugate forces. A fourth-order damage tensor *D_ijkl_* is used to characterize the anisotropic of the damage, as:

(6) $$D_{ijkl}\equiv \left[\begin{array}{cccccc} D_{1111} & D_{1122} & D_{1133} & D_{1112} & D_{1113} & D_{1123}\\ D_{2211} & D_{2222} & D_{2233} & D_{2212} & D_{2213} & D_{2223}\\ D_{3311} & D_{3322} & D_{3333} & D_{3312} & D_{3313} & D_{3323}\\ D_{1211} & D_{1222} & D_{1233} & D_{1212} & D_{1213} & D_{1223}\\ D_{1311} & D_{1322} & D_{1333} & D_{1312} & D_{1313} & D_{1323}\\ D_{2311} & D_{2322} & D_{2333} & D_{2312} & D_{2313} & D_{2323} \end{array}\right].$$

For the convenience of presentation, the damage tensor is replaced by a 6 × 6 matrix ***D***, as:

(7) $$D\equiv \left[\begin{array}{cccccc} D_{11} & D_{12} & D_{13} & 0 & 0 & 0\\ D_{21} & D_{22} & D_{23} & 0 & 0 & 0\\ D_{31} & D_{32} & D_{33} & 0 & 0 & 0\\ 0 & 0 & 0 & D_{44} & 0 & 0\\ 0 & 0 & 0 & 0 & D_{55} & 0\\ 0 & 0 & 0 & 0 & 0 & D_{66} \end{array}\right].$$

The nonlinear behavior is obtained by Helmholtz free energy *Ψ* expressions:

(8) $$\psi =\frac{1}{2\rho }\varepsilon :\left[C(D)+C^{t}(D)\right]:\varepsilon ,$$

where ***ε*** denotes the strain, and *ρ* represents the material density. ***C***(***D***) and ***C^t^***(***D***) are the initial modulus and tangential modulus, respectively.

Thus, the constitutive relationship can be given as:

(9) $$\sigma =\rho \frac{\partial \psi }{\partial \varepsilon }=\left[C(D)+C^{t}(D)\right]:\varepsilon ,$$

where ***σ*** stands the stress.

**Figure 5:**
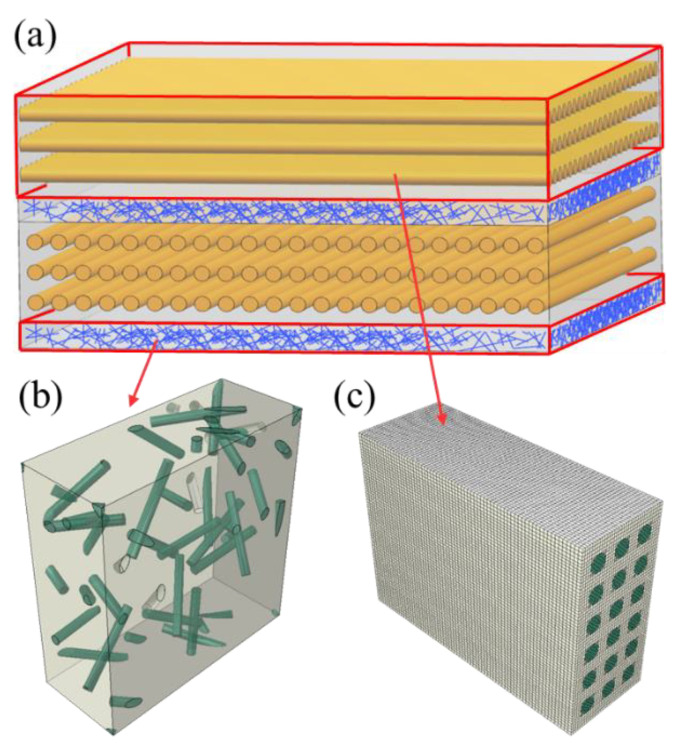
Geometric model of each component. (**a**) the un-needling region (**b**) short-cut fibers ply (**c**) weftless ply.

**Figure 6:**
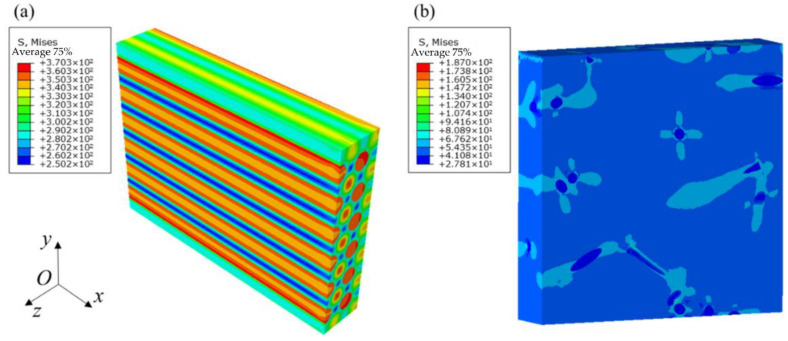
Mises stress contours for a shear strain *γ_yz_* of 1%. (Unit: MPa) (**a**) unidirectional fibers (**b**) short-cut fibers.

On the other hand, the CDM model requires the stiffness matrix as input. Since the un-needling region embraces both unidirectional and short-cut fiber material phases with a typical hierarchical structure, a hierarchical modelling approach is adopted [49,50], as shown in Figure 5. For the short-cut fiber plies, the random sequential adsorption (RSA) algorithm is implemented to generate the representative volume element (RVE) model [51], while the fiber-based RVE is applied for unidirectional fibers [52]. The differential method [53] is utilized to quantify the degree of matrix stiffness reduction by pores, yielding the stiffness of the matrix with pores as an input to the elastic constant calculation. The FE results of the equivalent elastic properties calculations are presented in Figure 6 and the predicted results are listed in Table 2. Due to the random orientation of the RSA-generated fibers, multiple models are created, with the average of the elastic constants calculated multiple times being applied as the final result for the short-cut fiber plies [54]. The unidirectional and short-cut fiber layers are simplified to homogeneous layers, followed by homogenization to obtain the elastic properties in the un-needling regions.

### 3.3. Multi-Scale Method

**Figure 7:**
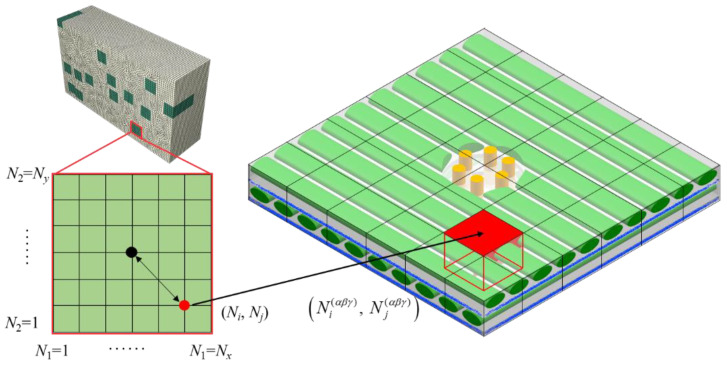
Local volume average of needling region mesoscopic damage.

The concept of multi-scale simulations is elaborated in this section. In order to establish a link between mesoscale and macroscale data of needling regions damage, a local volume average method is proposed. An integration point (marked as a red point in Figure 7) in the macroscopic model is presented below as an example.

1. The position sequence number of the integration point (*N_i_*, *N_j_*) is determined by the coordinate relationship with the center point of the needling hole;
2. The GMC model is divided into blocks to identify the corresponding local regions (marked as red rectangles in Figure 7) and subcell sequences of macroscopic model integration points;
3. The macroscopic field magnitude at (*N_i_*, *N_j_*) is obtained by volume average of the local region.

The relationship between the macroscopic stress ${\bar{\sigma }}_{ij}$, the strain ${\bar{\varepsilon }}_{ij}$ of the needling regions and the mesoscopic stress $\sigma_{ij}^{\left(\alpha \beta \gamma \right)}$, the strain $\varepsilon_{ij}^{\left(\alpha \beta \gamma \right)}$ of the subcell is as follows:

(10) $${\bar{\sigma }}_{ij}=\frac{1}{V}\sum_{\alpha =N_{i}^{\left(\alpha \beta \gamma \right)}-\delta }^{N_{i}^{\left(\alpha \beta \gamma \right)}+\delta }\sum_{\beta =N_{j}^{\left(\alpha \beta \gamma \right)}-\delta }^{N_{j}^{\left(\alpha \beta \gamma \right)}+\delta }\sum_{\gamma =1}^{N_{0\gamma }+N_{90\gamma }+2N_{S\gamma }}v_{\alpha \beta \gamma }\sigma_{ij}^{\left(\alpha \beta \gamma \right)}$$

(11) $${\bar{\varepsilon }}_{ij}=\frac{1}{V}\sum_{\alpha =N_{i}^{\left(\alpha \beta \gamma \right)}-\delta }^{N_{i}^{\left(\alpha \beta \gamma \right)}+\delta }\sum_{\beta =N_{j}^{\left(\alpha \beta \gamma \right)}-\delta }^{N_{j}^{\left(\alpha \beta \gamma \right)}+\delta }\sum_{\gamma =1}^{N_{0\gamma }+N_{90\gamma }+2N_{S\gamma }}v_{\alpha \beta \gamma }\varepsilon_{ij}^{\left(\alpha \beta \gamma \right)},$$

where *V* represents the volume of the mesoscopic local region corresponding to the macroscopic model integration point, and *v_αβγ_* denotes the subcell volume. $\left(N_{i}^{\left(\alpha \beta \gamma \right)},\;N_{j}^{\left(\alpha \beta \gamma \right)}\right)$ is the central subcell sequence of the mesoscopic local region, and 2*δ* represents the number of subcells in the local region.

The calculation of the mesoscopic stress can be expressed as [36]:

(12) $$\sigma^{\left(\alpha \beta \gamma \right)}=C^{\left(\alpha \beta \gamma \right)}\varepsilon^{\left(\alpha \beta \gamma \right)},$$

where ***C***^(*αβγ*)^ is the subcell stiffness matrix. At the same time, the subcell strain needs to be available through the macroscopic strain response [36]:

(13) $$\varepsilon^{\left(\alpha \beta \gamma \right)}=A^{\left(a\beta \gamma \right)}\bar{\varepsilon }\left(I+H^{\left(\alpha \beta \gamma \right)}\right),$$

where ***A***^(*αβγ*)^ and ***H***^(*αβγ*)^ denote the strain concentration coefficient matrix. The stress-strain relationship after volume average is achieved by coupling Equations (10), (12) and (13).

(14) $$\bar{\sigma }=\left[\frac{1}{V}\sum_{a=N_{i}^{\left(\alpha \beta \gamma \right)}-\delta }^{N_{i}^{\left(\alpha \beta \gamma \right)}+\delta }\sum_{\beta =N_{j}^{\left(\alpha \beta \gamma \right)}-\delta }^{N_{j}^{\left(\alpha \beta \gamma \right)}+\delta }\sum_{\gamma =1}^{N_{0\gamma }+N_{90\gamma }+2N_{S\gamma }}v_{a\beta \gamma }C^{\left(\alpha \beta \gamma \right)}A^{\left(\alpha \beta \gamma \right)}\left(I+H^{\left(a\beta \gamma \right)}\right)\right]\bar{\varepsilon }.$$

In summary, the main ideas of multi-scale simulation are as follows. Firstly, the strain increments at the integration points are given by macroscopic model calculations. Subsequently, the strain increments of subcells in the local region are calculated by Equation (13), which is used to solve the subcell stresses with Equation (12). In the next section, the subcell stiffness matrix is updated according to the damage criterion defined in Section 3.1, after which the damage matrix is determined based on the degradation of the stiffness matrix compared to the initial stiffness matrix. Finally, the macroscopic stress and stiffness matrices are updated using Equation (14), during which process the macroscopic damage of the needling region is evaluated.

## 4. Results and Discussions

The commercial FE software ABAQUS™ (V6.11, Beijing, China) [55] is utilized for macroscopic simulations, while the mesoscopic mechanical response of needling regions is calculated with a FORTRAN [56] coded program for GMC models. The user-defined material (UMAT) suboutline is used as the interface for macroscopic and mesoscopic data transfer. In-plane tensile load is applied to the macroscopic model. For any increment in ABAQUS™, the macroscopic strain of the needling regions at the integration point is passed through UMAT to the GMC program, where the mesoscopic simulation is performed and the volume average is transferred to ABAQUS™.

**Figure 8:**
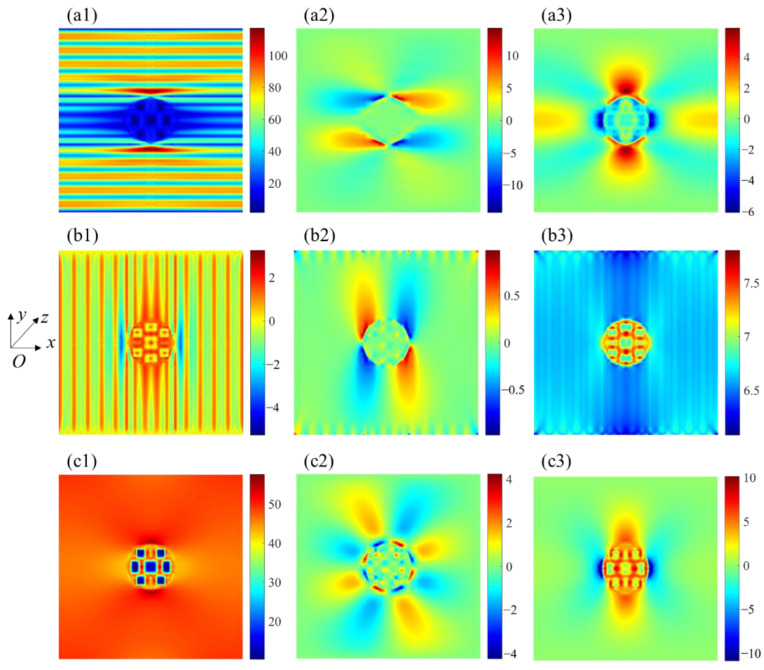
Stress contour for needling region: (**a1**) *σ*_11_, (**a2**) *σ*_12_ and (**a3**) *σ*_22_ for 0° unidirectional fibers; (**b1**) *σ*_11_, (**b2**) *σ*_12_ and (**b3**) *σ*_22_ for 90° unidirectional fibers; (**c1**) *σ*_11_, (**c2**) *σ*_12_ and (**c3**) *σ*_22_ for short-cut fibers (unit: MPa).

Figure 8 represents the stress distribution for each material phase at strain ${\bar{\varepsilon }}_{x}$ = 0.1%. The fiber breakage in the weftless ply due to the needling process caused a reduction of the in-plane load-bearing capacity in the needling hole. As can be observed in Figure 8a1–a3, a stress redistribution occurred around the needling hole, resulting in a stress concentration area at the fibers on both sides of the hole. The high-stress area is consistent with the experimental observations by Nie et al. [17]. When the fibers are perpendicular to the tensile direction, as shown in Figure 8b1–b3, the matrix stress is higher since the fibers cannot carry the load. Since the short-cut fibers are approximately in-plane isotropic materials, the stress distribution exhibits the same properties in Figure 8c1–c3.

**Figure 9:**
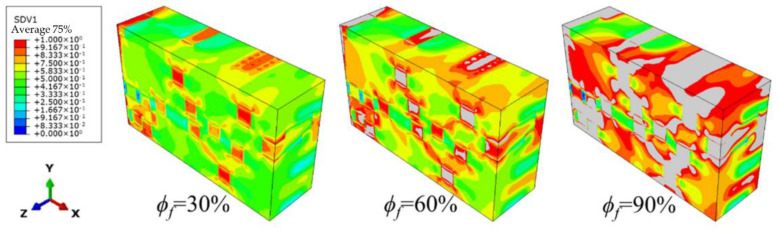
Damage evolution process in macroscopic model of 3D needled composites.

Macroscopic simulations of the needled composites are carried out by applying tensile loads in the *x*-direction, of which the damage evolution process is exhibited in Figure 9. The *ϕ_f_* is defined as the damage variable, *ϕ_f_* = ${\bar{\varepsilon }}_{x}$/${\bar{\varepsilon }}_{fx}$ where ${\bar{\varepsilon }}_{fx}$ is the failure strain. It aims to visually represent the correspondence between the contour and the damage level. The failure is presumed to occur when the damage at the material point exceeds one. During the process of tensile loading, the damage accumulates significantly faster in the needling regions than that in the un-needling regions, due to the stress concentration generated by the manufacturing defects. Thus, the failure occurs firstly in the needling regions, and then gradually become contiguous, leading to the structural failure.

**Figure 10:**
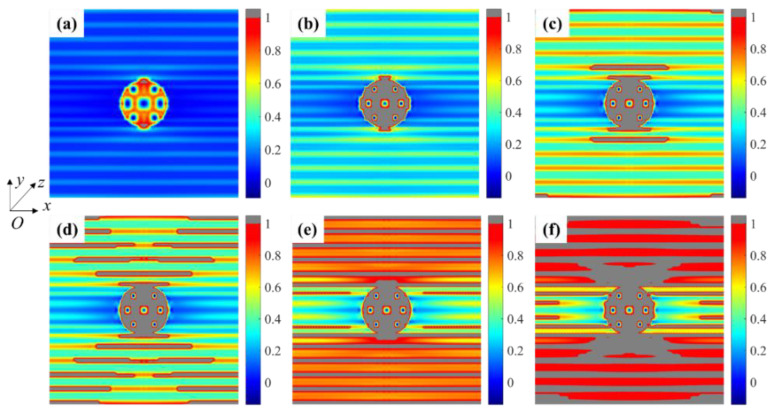
Damage evolution of the needling region in 0° unidirectional fiber ply (**a**) damage initiation (**b**) needling hole failure (**c**) matrix damage on both sides of the hole (**d**) failure of matrix between unidirectional fibers begins (**e**) complete failure of the matrix (**f**) fibers failure.

The damage process of the needling regions in the 0° weftless ply is displayed in Figure 10, where (a) to (f) indicate the different stages of damage initiation, propagation, and final failure. The damage is much higher in the needling holes than in continuous fiber parts owing to being carried only by the matrix. As shown in Figure 10a, b, the stress concentration areas on both sides fail first, followed by the matrix around the needling fibers. Afterwards, Figure 10c–e illustrate the rapid propagation of matrix cracks along the fiber direction. With the progressive failure of the matrix, the medium for transferring the load between the fibers is deprived. The fiber damage gradually accumulates as the load further increases, resulting in the failure of the needling hole, as depicted in Figure 10f.

**Figure 11:**
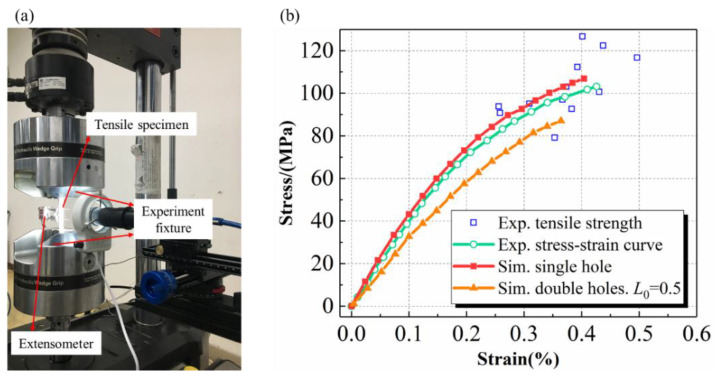
(**a**) The tensile specimens, experiment fixtures, and the extensometer (**b**) comparison of simulation and experimental results for tensile stress-strain curve [57].

The tensile experiment is then carried out, while the strain is measured by the extensometer. The loading rate of the machine chuck is 0.5 mm/min. The tensile specimens, experiment fixtures, and the extensometer are shown in Figure 11a. Experimental results show that the measured tensile strength and failure strain are 103.16 MPa and 0.426%, respectively. Figure 11b plots the in-plane tensile stress-strain curves obtained from the simulation (red line) and experiments (green line). The results are further compared with the test points (marked in blue) in Reference [46]. The relative error between predicted strength and experimental results is 2.6%, while the failure strain prediction error is about 4.4%. The high prediction is probably due to the presence of repeated needling in the same region. Nevertheless, the proposed method agrees well with the experimental results, providing a reasonable description of the nonlinear deformation behavior and failure limits for the needled composites.

**Figure 12:**
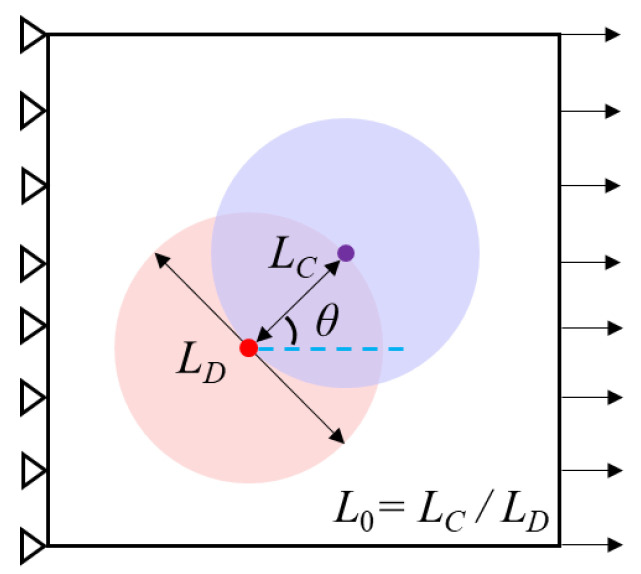
Schematic diagram of repeated needling region.

Considering the randomness and compactness of the needling location, there is a certain probability of repeated needling. Therefore, the damage process in the repeated needling region requires attention as well. A schematic diagram of the repeated needling region is given in Figure 12. Assuming that the repeated needling is performed twice, a partial overlap of the needling holes can be modeled. *L_D_* indicates the diameter of the needling hole, where *L_C_* is the distance between the centers of the two holes. The dimensionless length *L*_0_ is defined to quantify the overlapping size of the two holes. *L*_0_ = *L_C_*/*L_D_*, *L*_0_∈ [0, 1], where the upper and lower limits indicate complete overlap and separation of the two holes, respectively. The parameter *θ* represents the angle between the loading direction and the line connecting the two center points.

**Figure 13:**
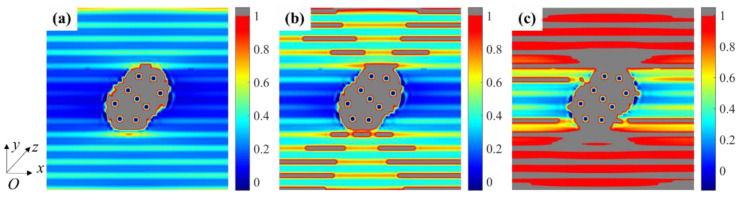
Damage evolution in the repeated needling region under tensile load (**a**) damage initiation and needling hole failure (**b**) failure of matrix between unidirectional fibers begins (**c**) fibers failure.

The damage process in the repeated needling region when *L*_0_ = 0.5 and *θ* = 45° is depicted in Figure 13, where (a), (b), and (c) indicate the stages of damage initiation, propagation, and final failure, respectively. The damage evolution is similar to that of a single one: the needling region fails first, leading to matrix cracking in the high stress area on both sides, which subsequently involves fiber fracture causing loss of load carrying capacity. However, the larger manufacturing defect of the double holes accelerates the damage accumulation rate. As seen in Figure 11b, the tensile strength, as well as the failure strain, obtained through the double-hole damage simulation (orange line) is significantly lower than that of a single hole. Such results indicate that multiple needling at the same location should be avoided as far as possible during the actual processing.

**Figure 14:**
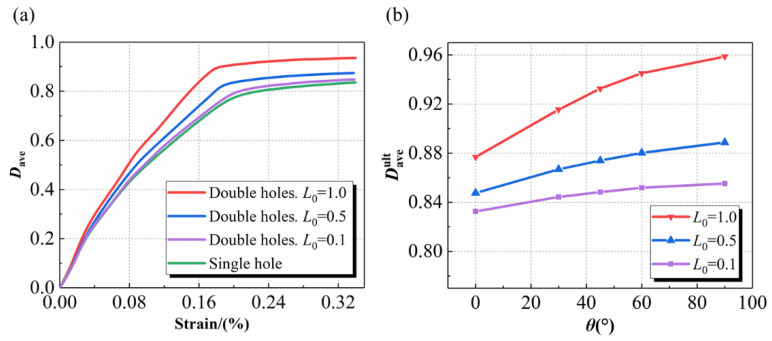
(**a**) Average damage at different *L*_0_ when *θ* = 45° (**b**) the variation law of the damage ultimate value with the angle *θ*.

Figure 14a illustrates the damage process at different *L*_0_, where *D*_ave_ denotes the volume-averaged damage of the needling region mesoscopic model. The damage curve represents a two-stage property: in the first stage, the damage accumulates rapidly, while the structure fails mostly after the turning point is exceeded; while in the second stage, the damage increases flatly. As the *L*_0_ increases, the distance between the two holes grows, meaning that the defect area becomes larger. The increased defect area also indicates an increased number of broken fibers, resulting in more severe stress concentrations in the needling regions. Therefore, it can be found that the larger the *L*_0_ the earlier the turning point appears, meaning that the sooner the structural failure occurs. The variation law of the failure value with *θ* is shown in Figure 14b, where $D_{\mathrm{ave}}^{\mathrm{ult}}$ indicates the damage value at failure. With the increase of *θ*, the more broken fibers caused by repeated needling, leading to an enlarged $D_{\mathrm{ave}}^{\mathrm{ult}}$ in the needling region.

## 5. Conclusions

In this paper, a geometric partitioning strategy is employed for the mechanical property’s simulation of 3D needled composites: the un-needling regions are characterized by the CDM model. Then, the GMC models containing needling holes and needling fibers are developed for different material phases in the needling regions, with multiscale damage quantification achieved by the local volume average method. Subsequently, the stress distribution and damage mechanism in the needling region are described. The numerical results of the proposed method are compared with experimental data to verify the accuracy. Finally, a series of simulations are carried out using a needling double-hole model to quantify the effect of repeated needling on damage evolution. The conclusions are summarized as follows:

1. Based on the μCT tomography tests, the local volume fractions of weftless ply and needling fibers around the needling holes are measured evenly to be 76.2% and 18.7%, respectively. Accordingly, the GMC models of three material phases, i.e., 0°/90° unidirectional fibers and short-cut fibers, are established. The Hashin criterion provides a good description of the failure mechanism in the needling region: stress concentrations firstly appear on both sides of the needling hole perpendicular to the loading direction, followed by the gradual failure of the needling hole and the matrix between the fibers. Eventually, the fibers fracture after the removal of the load transfer medium;
2. After conducting the geometric partitioning strategy, the needling regions are described by the multi-scale method, while the un-needling regions are covered through the CDM model. By using the established simulation strategy, the relative errors of the predictions are 2.6% for strength and 4.4% for failure strain in unidirectional tension compared with experimental results. Meanwhile, the proposed approach reveals the damage evolution law in which the initial damage emerges from the stress concentration in the needling region, leading to matrix cracking, fiber fracture, and ultimately structural failure;
3. The damage properties of the repeated needling regions are investigated. The damage mechanism of the repeated needling model is similar to that of the single-hole model, but with an enhanced degree of damage. Results show that the damage accumulation in the repeated needling region is related to the dimensionless overlapping size *L*_0_ and loading angle *θ*. An increase in *L*_0_ and *θ* resulted in a faster rate of damage accumulation. When *θ* = 45°, the degree of damage to the double hole is at most 20.2% (*L*_0_ = 1.0) more than the single hole model. Therefore, we recommend that repeated needling should be avoided as far as possible during the preparation of needled composites.

Furthermore, it should be mentioned that the above conclusions are based on needled composites made of alternating 0° and 90° carbon fiber weftless, short-cut fiber layers. Future work would focus on the assessment of the mechanical properties of needled composites with random distribution of single and multi-hole needled regions, to further provide the basis and direction for subsequent reliability analyses.

## Figures and Tables

**Table 1:** Material parameters.

Components	Parameter	Value
Carbon fiber [36]	Longitudinal modulus (GPa)	230
	Transverse modulus (GPa)	18.226
	Longitudinal Poisson’s ratio	0.27
	Transverse Poisson’s ratio	0.3
	Longitudinal Shear modulus (GPa)	36.597
	Transverse Shear modulus (GPa)	7.01
	Fiber volume fraction of weftless plies [25]	0.24
	Fiber volume fraction of short-cut fibers [25]	0.045
Matrix	Elastic modulus (GPa)	22.607
	Poisson’s ratio	0.218
Porosity	Pore volume fraction	0.0985

**Table 2:** Predicted material elastic properties (unit of elastic modulus and shear modulus: GPa).

	*E_x_*	*E_y_*	*E_z_*	*ν_xy_*	*ν_xz_*	*ν_yz_*	*G_xy_*	*G_xz_*	*G_yz_*
Weftless ply	81.45	21.69	21.69	0.231	0.231	0.268	13.06	13.06	5.54
short-cut fibers	20.59	20.37	19.65	0.215	0.221	0.216	7.96	7.52	6.85
un-needling region	59.93	59.93	20.02	0.230	0.251	0.255	14.27	11.01	10.95

## Data Availability

The data presented in this study are available on request from the corresponding author.
